# Epidemiology and prediction model of patients with carcinosarcoma in the United States

**DOI:** 10.3389/fpubh.2022.1038211

**Published:** 2022-11-28

**Authors:** Mingjing Chen, Xiandong He, Qiao Yang, Jia Zhang, Jiayi Peng, Danni Wang, Kexin Tong, Wenxiang Huang

**Affiliations:** ^1^Chongqing Key Laboratory of Infectious Diseases and Parasitic Diseases, Department of Infectious Diseases, The First Affiliated Hospital of Chongqing Medical University, Chongqing, China; ^2^Department of Thoracic Surgery, Daping Hospital, Army Medical University, Chongqing, China; ^3^Department of Ultrasound, The 941st Hospital of the People's Liberation Army Joint Logistic Support Force, Xining, China; ^4^Department of Geriatrics, The First Affiliated Hospital of Chongqing Medical University, Chongqing, China

**Keywords:** carcinosarcoma, SEER database, epidemiology, initial treatment, nomogram

## Abstract

**Background:**

Carcinosarcoma is a rare biphasic tumor composed of both carcinoma and sarcoma elements, which occurs at various sites. Most studies are case reports or small population-based studies for a single disease site, so comprehensive evaluations of epidemiology and prognostic factors for carcinosarcoma are needed.

**Methods:**

Surveillance, Epidemiology, and End Results (SEER)-8 (1975–2019) provided data for the epidemiological analysis. SEER-17 (2000–2019) provided data on the primary tumor sites, initial treatment, construction, and validation of the nomogram.

**Results:**

The age-adjusted incidence per 100,000 persons of carcinosarcoma increased significantly from 0.46 to 0.91 [1975–2019; average annual percent change (AAPC): 1.3%, *P* = 0.006], with localized stage increasing from 0.14 to 0.26 [2005–2015; annual percent change (APC): 4.2%]. The 20-year limited-duration prevalence per 100,000 increased from 0.47 to 3.36 (1999–2018). The mortality per 100,000 increased significantly from 0.16 to 0.51 (1975–2019; AAPC: 1.9%, *P* < 0.001). The 5-year relative survival was 32.8%. The greatest number of carcinosarcomas were from the uterus (68.7%), ovary (17.8%), lung and bronchus (2.3%). The main treatment is comprehensive treatment based on surgery; however, surgery alone is preferred in older patients. In multivariate analysis (*N* = 11,424), age, sex, race, year of diagnosis, disease stage, tumor site, and treatment were associated with survival. A nomogram was established to predict 1-, 3-, and 5-year survival, and the C-indexes were 0.732 and 0.748 for the training and testing sets, respectively. The receiver operating characteristic curve demonstrated that the nomogram provided a comprehensive and accurate prediction [1-year area under the curve (AUC): 0.782 vs. 0.796; 3-year AUC: 0.771 vs. 0.798; 5-year AUC: 0.777 vs. 0.810].

**Conclusions:**

In this study, the incidence, prevalence, and mortality of carcinosarcoma have increased over the past decades. There was a rapid rise in the incidence of localized stage in recent years, which reflected improved early detection. The prognosis of carcinosarcoma remains poor, signifying the urgency of exploring targeted cancer control treatments. Explicating distribution and gender disparities of carcinosarcoma may facilitate disease screening and medical surveillance. The nomogram demonstrated good predictive capacity and facilitated clinical decision-making.

## Introduction

Carcinosarcoma is a rare biphasic tumor composed of both carcinoma and sarcoma elements; this is the most lethal malignancy (1–3). Carcinosarcoma occurs at various sites, mainly in the uterus and ovary, but also in the lung, bladder, peritoneum, and gallbladder (4–9). Due to the highly aggressive behavior of carcinosarcomas, advanced stage at diagnosis and frequent recurrences may explain the poor prognosis (1, 5, 9).

Most studies on carcinosarcoma focused on gynecological carcinosarcoma and suggested that the incidence of carcinosarcoma gradually increased in recent years (5, 10). The vast majority of studies are case reports or small population-based studies for a single disease site (11–14). Previous researches on single pathological types indicated that primary tumor site could affect prognosis (15–17). In multisite tumors (MDM2-amplified liposarcoma, neuroendocrine tumor, undifferentiated multitype sarcoma, cutaneous melanoma), tumor site was a significant variable in survival prediction model (18–21). A study on liposarcoma proposed that anatomic localization and histological grade, and not tumor size, should be included in liposarcoma-specific staging system (18). Hence, it is necessary to explore the effect of tumor site on prognosis, especially for rare tumors. To date, there is insufficient systematic studies on carcinosarcoma based on large sample sizes. Distribution and prognosis of tumor site remain unclear. Using the Surveillance, Epidemiology, and End Results (SEER) program, we performed a large population and multi-primary tumor site epidemiological and clinical analysis of carcinosarcoma to provide more clinical evidence.

Because of the rarity and aggressiveness of carcinosarcoma, clinical trials are difficult to conduct, and comprehensive analysis of treatments is lacking; hence, there is no standard consensus for treatment guidelines (4, 9, 22, 23). The prognosis of carcinosarcoma is difficult to judge because of its complexity (5, 9). Therefore, this study analyzed treatment strategies and established a nomogram combining various factors to predict the survival probability based on a large population of patients with carcinosarcoma.

## Methods

### Data source

The data were obtained from the SEER database (www.seer.cancer.gov) of the National Cancer Institute using the SEER^*^Stat software (SEER^*^Stat 8.4.0). The SEER 8 registries program is a unique record for long-term (1975–2019) incidence, prevalence, mortality, and relative survival, representing about 8.3% of the US population, released April 2022. Analysis of ten leading specific tumor sites, initial treatment, as well as construction and validation of nomogram were based on the SEER 17 registries program (2000–2019); covering ~26.5% of the US population, released April 2022.

### Study population and variables

Carcinosarcoma (8,950/3, 8,951/3, 8,980/3, 8,981/3) was identified according to the International Classification of Diseases for Oncology, 3rd edition (ICD-O-3). Meanwhile, diagnoses were confirmed by positive histology; only one primary and the type of reporting source was not autopsy or death certificate.

The variables included age, sex, race, year of diagnosis, disease stage, site record, treatment, survival time, and vital status (alive/dead). The year of diagnosis was divided into four periods, including 2000–2004, 2005–2009, 2010–2014, and 2014–2019. Vital status was used as study endpoint, defined as any patient who died after the follow-up cut-off date was recorded to alive as of the cut-off date. Overall survival (OS) is defined as the period from the date of diagnosis to death. In the epidemiology section, the disease stage was referenced to SEER historic stage A (1975–2015); in the specific patient list, the disease stage was a combination of SEER historic stage A (1975–2015) and combined summary stage (2016–2019). Treatment was divided into no treatment/unknown; single treatment, chemotherapy; single treatment, radiotherapy; single treatment, surgery; chemotherapy + radiotherapy; chemotherapy + surgery; radiotherapy + surgery; and chemotherapy + radiotherapy + surgery by calculation.

### Study design

**Figure 1:**
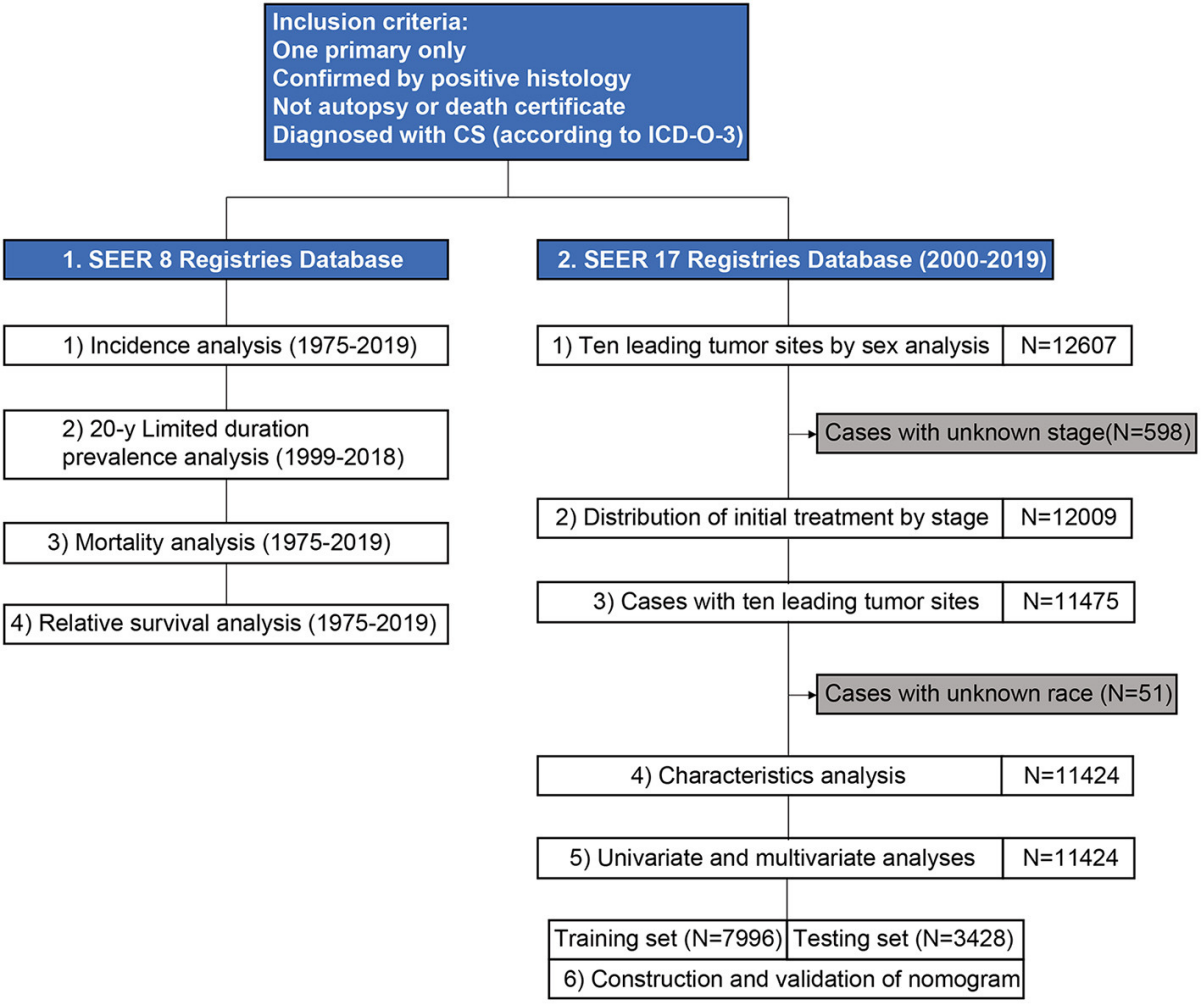
A flowchart of study design and patient selection. CS, carcinosarcoma; ICD-O-3, International Classification of Disease for Oncology, 3rd edition; SEER, surveillance, epidemiology, and end results.

The study design is presented in a flowchart (Figure 1). The age-adjusted incidence, prevalence, and age-adjusted mortality of carcinosarcoma were obtained from the SEER 8 database and expressed per 100,000 persons, referring to the 2,000 US standard population. The trends of incidence and mortality, including the annual percent change (APC) and average annual percent change (AAPC), were quantified using the Joinpoint Regression Program (version 4.7), allowing up to two joinpoints. The last 20-year prevalence and the 1-, 3-, and 5-year relative survival for carcinosarcoma were calculated using the SEER^*^Stat 8.4.0.

A detailed patient list was obtained from SEER 17. Ten leading specific tumor sites by sex were analyzed, and the distribution of initial treatment by stage and age was assessed. This study included patients with carcinosarcoma of the ten leading specific tumor sites. Patient characteristics were presented, and univariate and multivariate analysis were performed to investigate prognostic factors associated with survival. Patients were randomly divided into training and testing sets at a ratio of 7:3. A nomogram to predict 1-, 3-, and 5-year survival for carcinosarcoma was constructed based on the prognostic factors in the training set, and concordance indexes (C-index) and area under the curve (AUC) were used to evaluate the nomogram.

### Statistical analysis

Variables were presented as frequency and percentage and were compared using Pearson's chi-square test. Univariate and multivariate Cox proportional hazards models were used to assess the prognostic factors associated with OS by calculating the hazard ratio (HR) and 95% confidence interval (CI). C-index and calibration curves were used to evaluate the discriminative ability in the training and testing sets. The total nomogram score for each patient was obtained, and the corresponding AUC was used to estimate accuracy. Statistical analyses were performed using the R software (version 4.0.5; http://www.r-project.org/). All tests of statistical significance were 2-sided, and a *P*-value < 0.05 was considered statistically significant.

## Results

### Annual incidence

**Figure 2:**
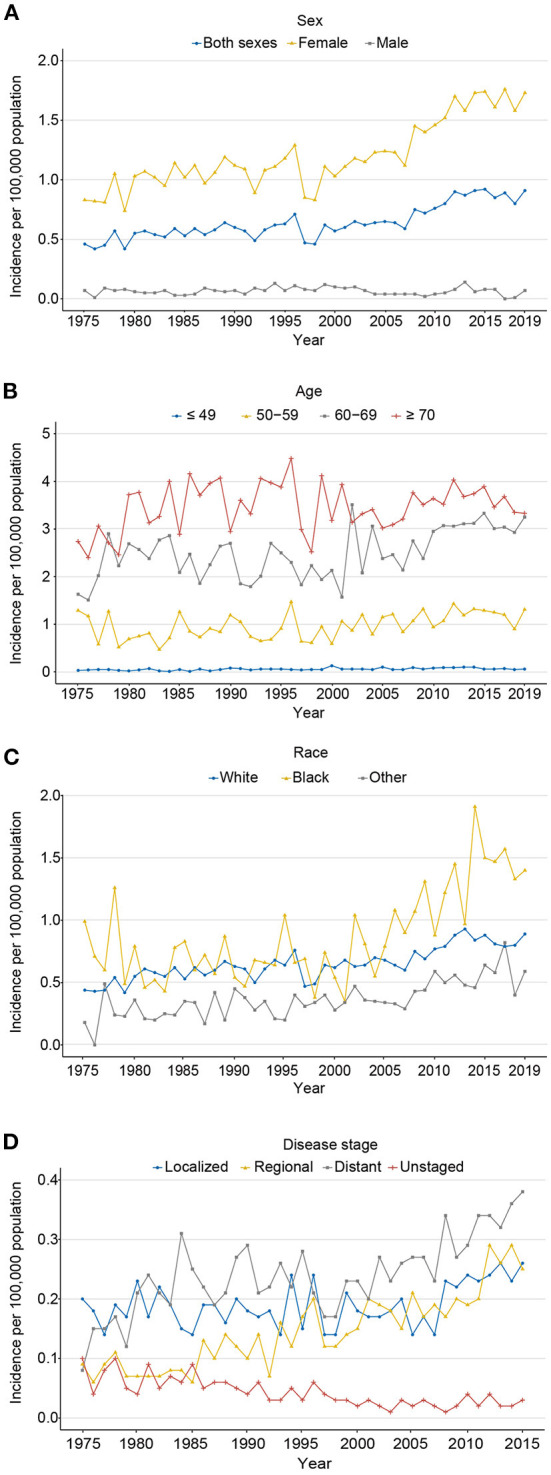
The age-adjusted incidence of carcinosarcoma, SEER-8. **(A)** for sex (1975–2019); **(B)** for age (1975–2019); **(C)** for race (1975–2019); and **(D)** for disease stage (1975–2015).

Using population data obtained from SEER 8, the age-adjusted incidence of all (both sexes) cases showed a marked increase from 0.46 in 1975 to 0.91 per 100,000 persons in 2019 (AAPC: 1.3%, *P* = 0.006), largely because of a rapid increase among cases in women (AAPC: 1.5%, *P* < 0.001) rather than in men (AAPC: 0.0%, *P* = 0.986; 1.73 vs. 0.07 per 100,000 persons in 2019, respectively; Figure 2A). As for age at diagnosis, the incidence was highest in ≥70, followed by 60–69, 50–59, and ≤ 49 years (3.33 vs. 3.25 vs. 1.31 vs. 0.06 per 100,000 persons in 2019), with an AAPC of 0.7% (*P* = 0.143), 1.9% (*P* = 0.073), 0.9% (*P* = 0.003), and 1.6% (*P* = 0.001), respectively (Figure 2B). The incidence rates of white, black, and other races were rising over the past decades, with an AAPC of 1.3% (P < 0.001), 1.8% (*P* = 0.005), and 2.5% (*P* < 0.001), respectively. Among black populations, a marked increase in incidence was observed from 1998 to 2019 (0.38–1.40 per 100,000 persons), with an APC of 4.7% (Figure 2C). From 1975 to 2015, the incidence of localized (AAPC: 0.9%, *P* = 0.045), regional (AAPC: 3.3%, *P* < 0.001), and distant stages (AAPC: 1.9%, *P* < 0.001) had a rising trend; among them, localized stage had increased the most in recent years (2005–2015, APC 4.2%). Patients with unknown stage decreased from 1975 to 2015, with an AAPC of −3.3% (*P* < 0.001; Figure 2D). Detailed data are shown in eTables 1, 2.

### Twenty-year limited-duration prevalence

**Figure 3:**
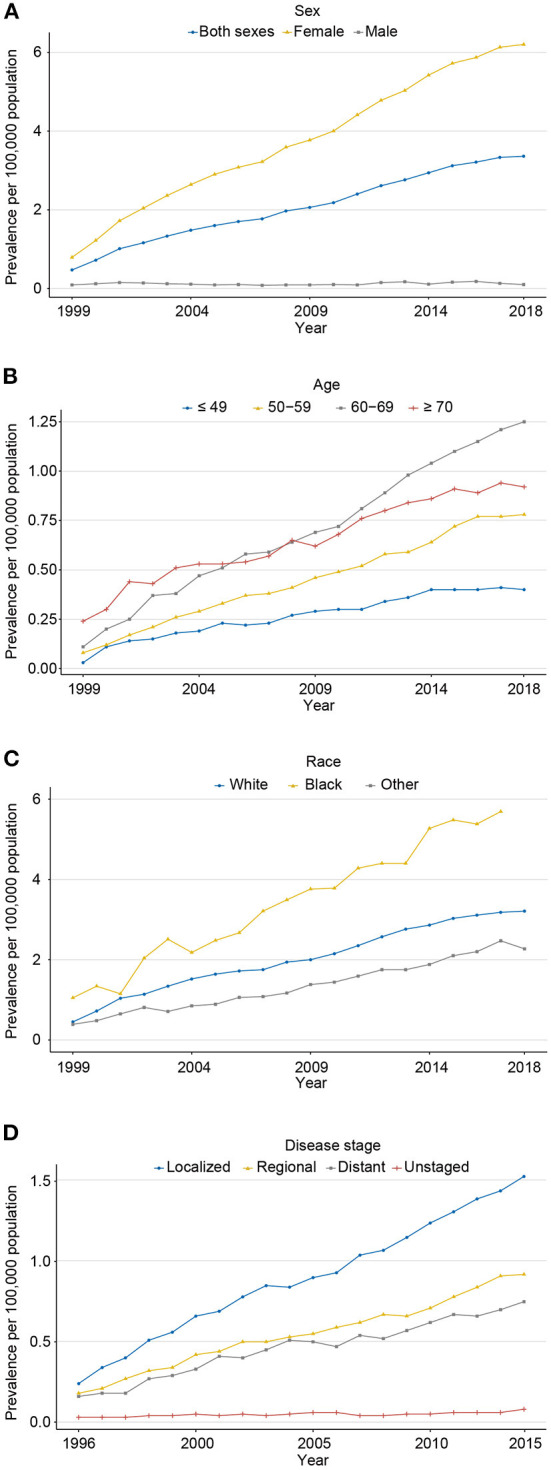
The 20-year limited-duration prevalence of carcinosarcoma, SEER-8. **(A)** for sex (1999–2018); **(B)** for age (1999–2018); **(C)** race (1999–2018); and **(D)** disease stage (1996–2015).

The 20-year limited-duration prevalence of all carcinosarcomas increased from 0.47 to 3.36 per 100,000 persons from 1999 to 2018 (Figure 3A; eTable 3). The prevalence in women was dramatically higher than that in men (6.20 vs. 0.10 per 100,000 persons in 2018). For age groups, the prevalence in 60–69 years was the highest, followed by ≥70, 50–59, and ≤ 49 years (1.25 vs. 0.92 vs, 0.78 vs, 0.40 per 100,000 persons in 2018, respectively; Figure 3B). Among different races, prevalence increased the most in the black population from 1.05 to 6.26 per 100,000 persons from 1999 to 2018, followed by white and other races (Figure 3C). For stage groups, the prevalence increased most in localized, followed by regional, distant, and unknown stages (1.53 vs. 0.92 vs. 0.75 vs. 0.08 per 100,000 persons in 2015, respectively; Figure 3D).

### Annual mortality

**Figure 4:**
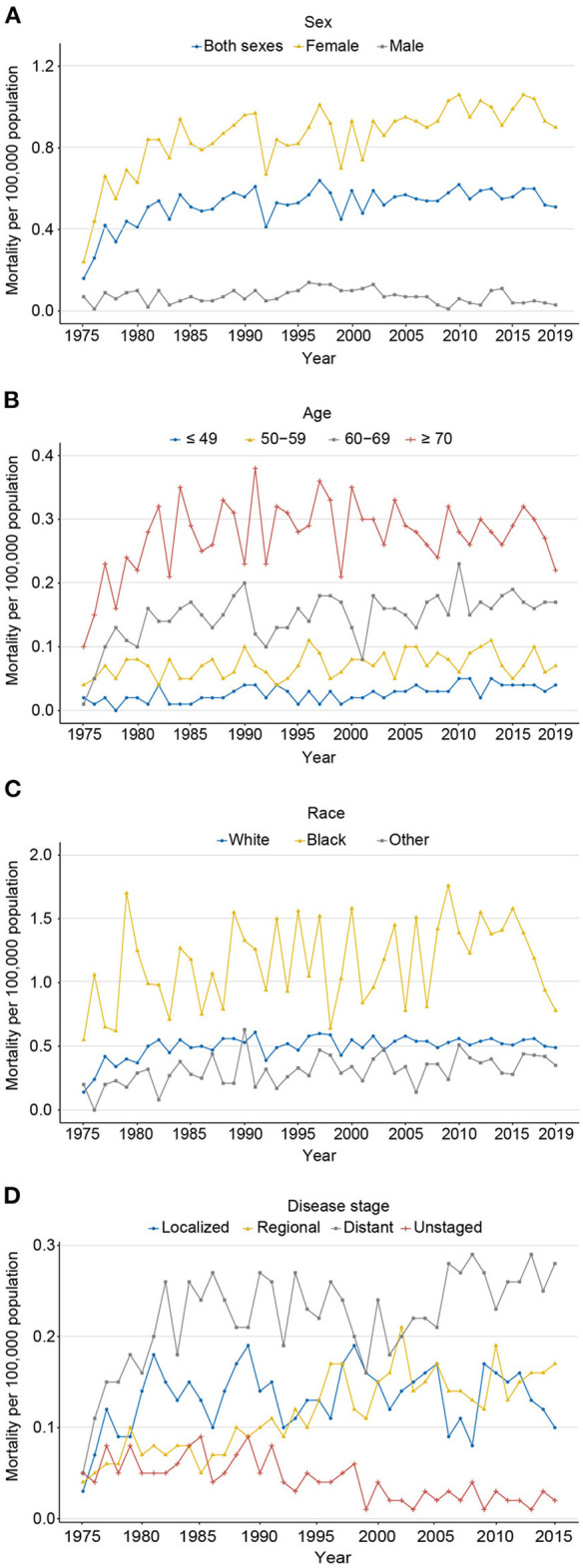
The age-adjusted mortality of carcinosarcoma, SEER-8. **(A)** for sex (1975–2019); **(B)** for age (1975–2019); **(C)** for race (1975–2019); and **(D)** for disease stage (1975–2015).

Contrary to the rapid progress against cancer, the mortality of carcinosarcoma increased significantly from 0.16 to 0.51 per 100,000 persons from 1975 to 2019, with an AAPC of 1.9% (*P* < 0.001; Figure 4A; eTables 4, 5). The mortality rate for women still increased from 2015 to 2019, with an AAPC of 0.5% (*P* = 0.001); fortunately for men, it decreased sharply, with an AAPC of −4.1% (*P* = 0.003). Patients aged 70 years or older had the highest death rate, followed by 60–69, 50–59, and ≤ 49 years (0.22 vs. 0.17 vs. 0.07 vs. 0.04 per 100,000 persons in 2019, respectively), with an AAPC of 1.6% (*P* = 0.022), 5.6% (*P* = 0.120), 0.8% (*P* = 0.013), and 2.0% (*P* < 0.001), respectively (Figure 4B). The mortality rate in black populations edged up from 0.55 in 1975 to 0.78 per 100,000 persons in 2019, with an AAPC of −0.5% (*P* = 0.524; Figure 4C). Meanwhile, steady increases in mortality for white and other races were observed from 1975 to 2019, with an AAPC of 3.0% (*P* = 0.036) and 1.3% (*P* = 0.029), respectively. Notably, from 1975 to 2019, mortality only declined in unknown stage (AAPC −3.4%, *P* < 0.001), stabilized at localized stage (AAPC 2.1%, *P* = 0.124), and increased in regional (AAPC 2.9%, *P* < 0.001) and distant stages (AAPC 2.9%, *P* < 0.001; Figure 4D).

### Relative survival analysis

**Figure 5:**
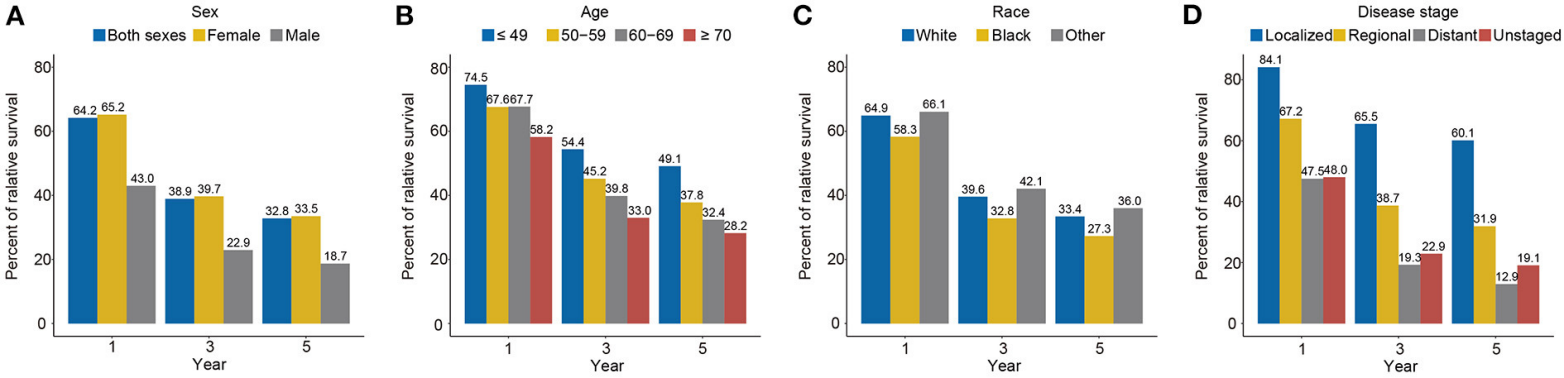
The 1-, 3-, and 5-year relative survival rates of carcinosarcoma by sex (**A**, 1975–2019), age (**B**, 1975–2019), race (**C**, 1975–2019), and disease stage (**D**, 1975–2015), SEER-8.

The 1-, 3-, and 5-year relative survival rate for all carcinosarcomas based on SEER 8 from 1975 to 2019 were 64.2, 38.9, and 32.8%, respectively (Figure 5). Survival rates were close to overall for women (1-year, 65.2%; 3-year, 39.7%; 5-year, 33.5%) and much worse for men (1-year, 43.0%; 3-year, 22.9%; 5-year, 18.7%). For age groups, survival rate was highest for ≤49 years (1-year, 74.5%; 3-year, 54.4%; 5-year, 49.1%) and lowest for ≥70 years (1-year, 58.2%; 3-year, 33.0%; 5-year, 28.2%). Among different races, black patients (1-year, 58.3%; 3-year, 32.8%; 5-year, 27.3%) had the worst prognosis than white (1-year, 64.9%; 3-year, 39.6%; 5-year, 33.4%) and other races (1-year, 66.1%; 3-year, 42.1%; 5-year, 36.0%). Survival was the best in the localized stage (1-year, 84.1%; 3-year, 65.5%; 5-year, 60.1%), followed by the regional stage (1-year, 67.2%; 3-year, 38.7%; 5-year, 31.9%), and the worst in the distant stage (1-year, 47.5%; 3-year, 19.3%; 5-year, 12.9%).

### Ten leading specific tumor sites by sex

**Figure 6:**
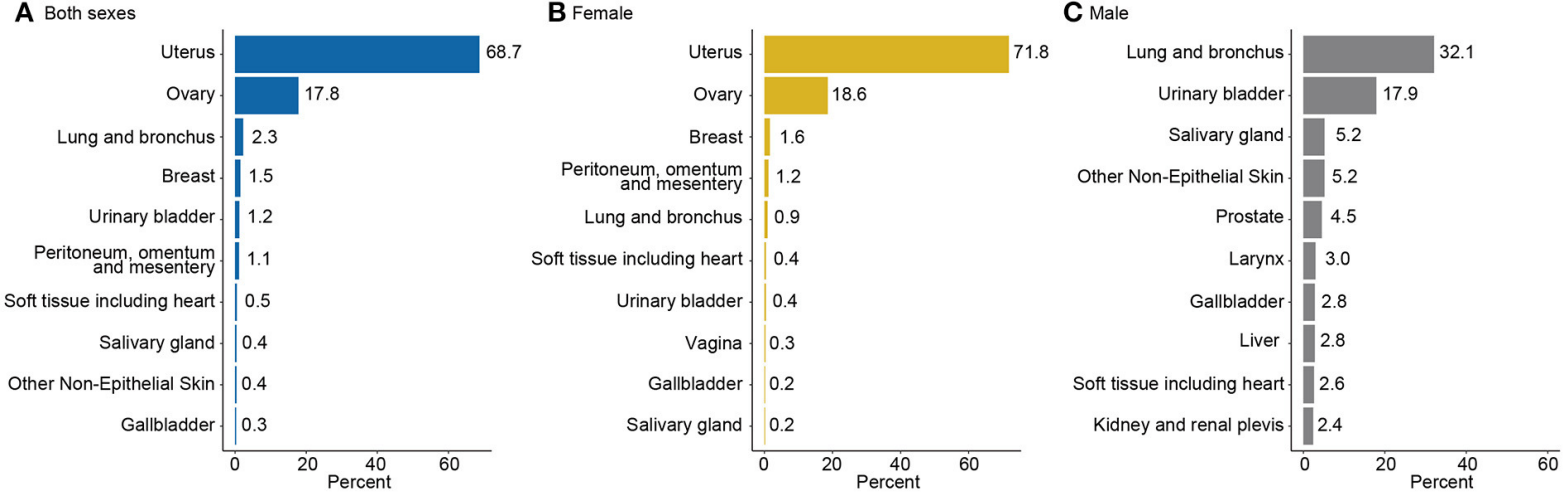
Ten leading specific tumor sites of carcinosarcoma by sex, SEER-17, 2000–2019. **(A)** Both sexes, **(B)** for female, and **(C)** for male.

Using a detailed patient list from SEER 17, Figure 6 presents the ten leading specific tumor sites for carcinosarcoma by sex. Of 12,607 cases, the greatest number of carcinosarcomas were from the uterus (68.7%), ovary (17.8%), lung and bronchus (2.3%), breast (1.5%), and urinary bladder (1.2%). In total, uterus, ovary, and breast accounted for 88.0% of all cases (Figure 6A). In women, uterus (71.8%), ovary (18.6%), and breast (1.6%) accounted for 92.0% of all female carcinosarcomas (Figure 6B). In men, lung and bronchus (32.1%), urinary bladder (17.9%), and salivary gland (5.2%) were the three most common sites (Figure 6C).

### Initial treatment for carcinosarcoma by stage and age

**Figure 7:**
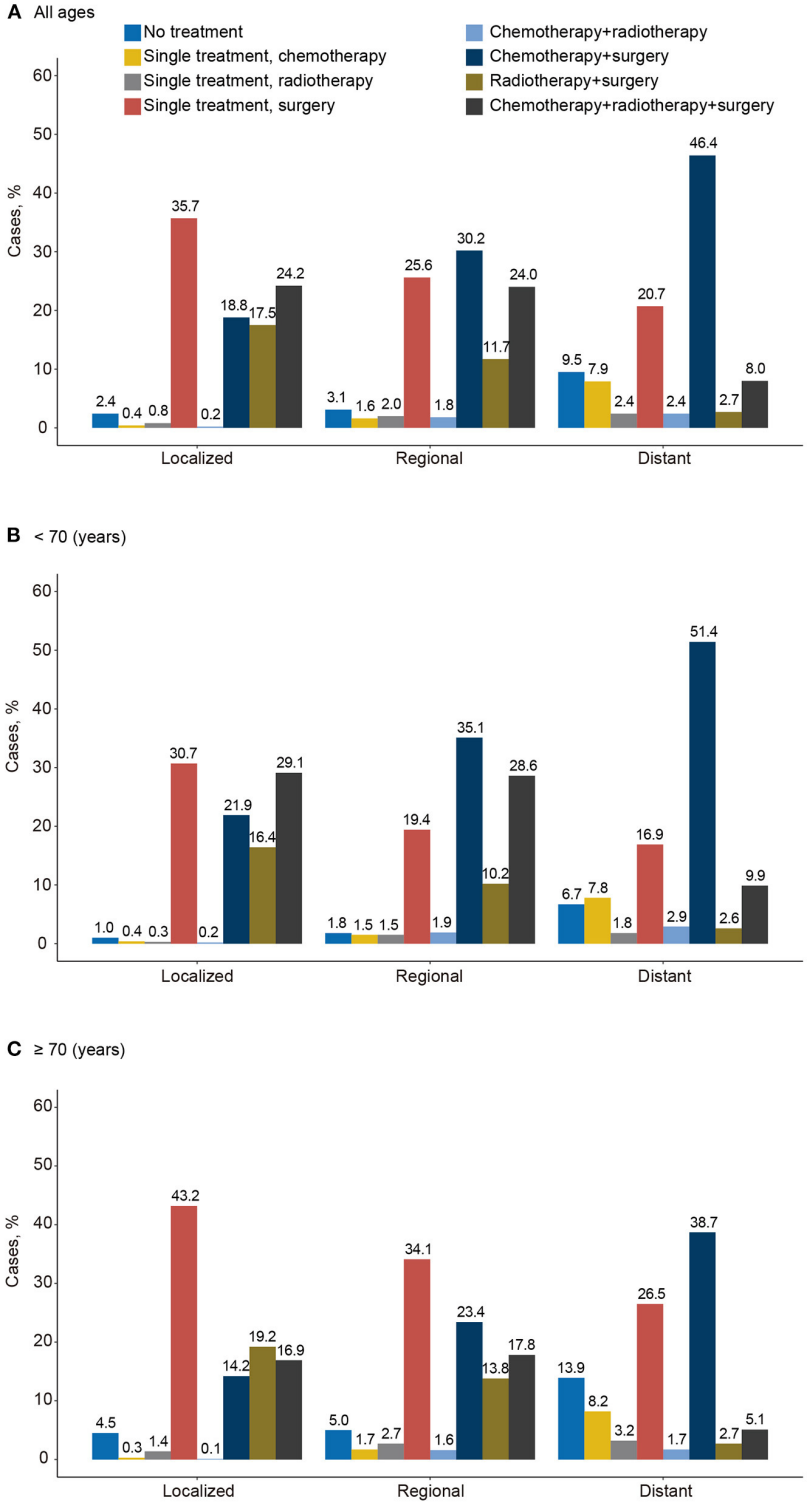
Distribution of initial treatment of carcinosarcoma by disease stage and age, SEER-17, 2000–2019. **(A)** All ages, **(B)** for <70 years, and **(C)** for ≥70 years.

From 2000 to 2019, 2.4% of localized, 3.1% of regional, and 9.5% of distant stage carcinosarcoma cases (12,009) were classified as receiving no treatment/unknown (Figure 7A). Receiving single surgery or surgery combined with other comprehensive modality treatments (including chemotherapy and radiotherapy) played the most important role in all stages of all carcinosarcomas (localized, 96.2%; regional, 91.5%; distant, 77.8%). In contrast, only a minority of the patients received a single treatment. The proportion of receiving no treatment/unknown was higher in the older patients (≥70 years) than in younger patients (<70 years) at every stage (localized, 4.5 vs. 1.0%; regional, 5.0 vs. 1.8%; distant, 13.9 vs. 6.7%; Figures 7B,C). A higher proportion of older patients received single surgery treatment than younger patients (localized, 43.2 vs. 30.7%; regional, 34.1 vs. 19.4%; distant, 26.5 vs. 16.9%), whereas a lower proportion of older than younger patients received surgery combined with chemotherapy and/or radiotherapy (localized, 31.1 vs. 51.0%; regional, 41.2 vs. 63.7%; distant, 43.8 vs. 61.3%).

### Patient characteristics, univariate and multivariate analyses

A total of 11,424 patients with carcinosarcoma of the ten leading specific tumor sites were identified in the following analysis (Table 1). Among these patients, 40.6% (4,639) were aged 70 years or older. Most of the patients were women (11,088, 97.1%) and white population (8,290, 72.6%). For year of diagnosis, 18.4% (2,105) were diagnosed in 2000–2004, 22.6% (2,580) were diagnosed in 2005–2009, 27.7% (3,161) were diagnosed in 2010–2014, and 31.3% (3,578) were diagnosed in 2015–2019. As for disease stage, 3,499 (30.6%) were localized, 3,453 (30.2%) were regional, and 4,472 (39.1%) were distant stage. Furthermore, the uterus (8,317, 72.8%) was the most common primary tumor site of carcinosarcoma, followed by ovary (2,187, 19.1%) and lung and bronchus (280, 2.5%). This was generally consistent with previous rankings. Receiving surgical treatment (3,048, 26.7%) or surgery combined with chemotherapy, radiotherapy, or chemoradiotherapy (7,016, 61.5%) dominated the treatment modalities.

**Table 1:** Baseline clinicopathological characteristics for all carcinosarcoma, training set, and testing set.

**Characteristic**	**All**	**Cohort**		***P***
		**Training set**	**Testing set**	
**Age (year)**				
≤ 49	779 (6.8)	227 (6.6)	552 (6.9)	0.801
50–59	2,109 (18.5)	624 (18.2)	1,485 (18.6)	
60–69	3,897 (34.1)	1,190 (34.7)	2,707 (33.9)	
≥70	4,639 (40.6)	1,387 (40.5)	3,252 (40.7)	
**Sex**				
Male	336 (2.9)	88 (2.6)	248 (3.1)	0.136
Female	11,088 (97.1)	3,340 (97.4)	7,748 (96.9)	
**Race**				
White	8,290 (72.6)	2,528 (73.7)	5,762 (72.1)	0.160
Black	2,268 (19.9)	657 (19.2)	1,611 (20.1)	
Other	866 (7.6)	243 (7.1)	623 (7.8)	
**Year of diagnosis**
2000–2004	2,105 (18.4)	1,474 (18.4)	631 (18.4)	0.741
2005–2009	2,580 (22.6)	1,808 (22.6)	772 (22.5)	
2010–2014	3,161 (27.7)	2,232 (27.9)	929 (27.1)	
2015–2019	3,578 (31.3)	2,482 (31.0)	1,096 (32.0)	
**Disease stage**				
Localized	3,499 (30.6)	1,068 (31.2)	2,431 (30.4)	0.418
Regional	3,453 (30.2)	1,007 (29.4)	2,446 (30.6)	
Distant	4,472 (39.1)	1,353 (39.5)	3,119 (39.0)	
**Primary tumor site**				
Uterus	8,317 (72.8)	2,547 (74.3)	5,770 (72.2)	0.313
Ovary	2,187 (19.1)	630 (18.4)	1,557 (19.5)	
Lung and bronchus	280 (2.5)	74 (2.2)	206 (2.6)	
Breast	186 (1.6)	57 (1.7)	129 (1.6)	
Urinary bladder	141 (1.2)	34 (1.0)	107 (1.3)	
Peritoneum, omentum, and mesentery	131 (1.1)	40 (1.2)	91 (1.1)	
Soft tissue including heart	59 (0.5)	15 (0.4)	44 (0.6)	
Salivary gland	46 (0.4)	13 (0.4)	33 (0.4)	
Other non-epithelial skin	38 (0.3)	11 (0.3)	27 (0.3)	
Gallbladder	39 (0.3)	7 (0.2)	32 (0.4)	
**Treatment**				
No treatment/unknown	582 (5.1)	186 (5.4)	396 (5.0)	0.383
Single treatment, chemotherapy	415 (3.6)	108 (3.2)	307 (3.8)	
Single treatment, radiotherapy	198 (1.7)	59 (1.7)	139 (1.7)	
Single treatment, surgery	3,048 (26.7)	921 (26.9)	2,127 (26.6)	
Chemotherapy + radiotherapy	165 (1.4)	58 (1.7)	107 (1.3)	
Chemotherapy + surgery	3,777 (33.1)	1,125 (32.8)	2,652 (33.2)	
Radiotherapy + surgery	1,152 (10.1)	358 (10.4)	794 (9.9)	
Chemotherapy + radiotherapy + surgery	2,087 (18.3)	613 (17.9)	1,474 (18.4)	

Univariate and multivariate analyses were performed to investigate prognostic factors associated with survival. In univariate analysis, age, sex, race, year of diagnosis, disease stage, primary tumor site and treatment were all statistically significant (eTable 6). In multivariate analysis, the disease stage and treatment were the most relevant prognostic factors (eTable 6). Compared with localized, the significant increase in mortality was observed in distant (HR: 4.57, 95% CI: 4.26–4.90; *P* < 0.001) and regional stages (HR: 2.41, 95% CI: 2.26–2.58; *P* < 0.001). All treatment modalities were effective, with triple therapies (HR: 0.13, 95% CI: 0.11–0.14; *P* < 0.001) being the most effective factor in improving OS. Compared with the uterus, the gallbladder (HR: 1.57, 95% CI: 1.09–2.27; *P* = 0.016) and soft tissues including heart (HR: 1.45, 95% CI: 1.09–1.94; *P* = 0.012) were associated with inferior survival. Other non-epithelial skin (HR: 0.52, 95% CI: 0.34–0.80; *P* = 0.003), salivary gland (HR: 0.56, 95% CI: 0.37–0.85; *P* = 0.007), and ovary (HR: 0.78, 95% CI: 0.73– 0.83; *P* < 0.001) were associated with superior survival. The other sites showed no significant differences. In addition, age (50–59 vs. 0–49 years: HR: 1.34, 95% CI: 1.19–1.49, *P* < 0.001; 60–69 vs. 0–49 years: HR: 1.49, 95% CI: 1.34–1.65, *P* < 0.001; ≥70 vs. 0–49 years: HR: 1.86, 95% CI: 1.68–2.07, *P* < 0.001), sex (female vs. male: HR: 0.70, 95% CI: 0.58–0.85, *P* < 0.001), race (black vs. white: HR: 1.17, 95% CI: 1.10–1.24; *P* < 0.001), and year of diagnosis (2005–2009 vs. 2000–2004: HR: 0.90, 95% CI: 0.84–0.96, *P* < 0.001; 2010–2014 vs. 2000–2004: HR: 0.88, 95% CI: 0.83–0.94, *P* < 0.001; 2015–2019 vs. 2000–2004: HR: 0.87, 95% CI: 0.81–0.93, *P* < 0.001) were significantly associated with survival.

### Construction and validation of nomogram

**Figure 8:**
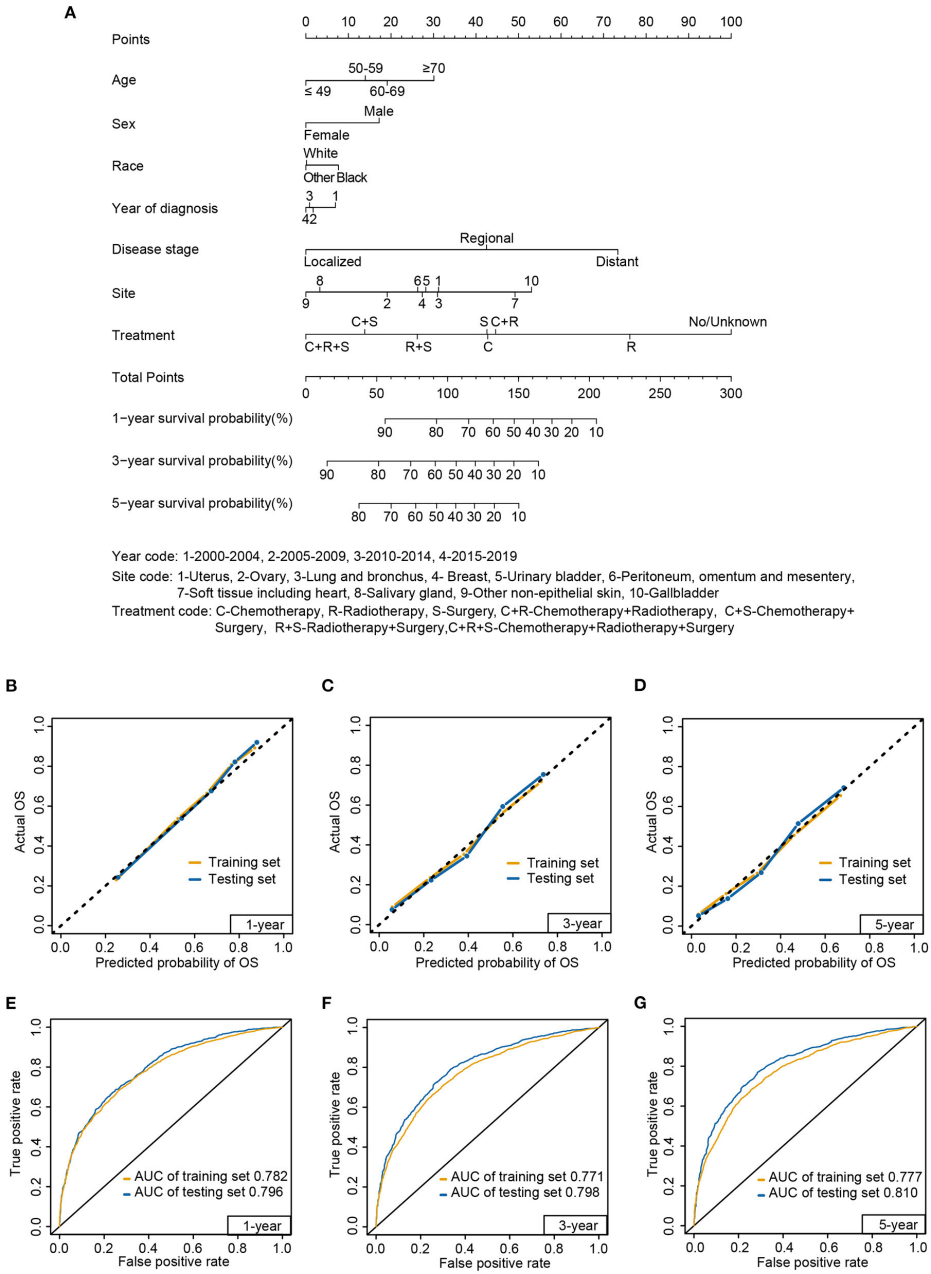
Nomogram **(A)** to predict 1-, 3-, and 5-year survival probabilities for carcinosarcoma, calibration curve **(B–D)**, and receiver operating characteristic **(E–G)** curve of the nomogram in the training set and testing set. AUC, area under the curve.

At a ratio of 7:3, patients were randomly assigned to the training (7,996) and testing sets (3,428, Table 1). There were no significant differences between the two sets. A nomogram for predicting 1-, 3-, and 5-year survival probability was constructed by including prognostic factors in the multivariate analysis based on the training set (Figure 8A). Consistent with previous results, treatment was the most significant factor associated with survival, followed by disease stage and primary tumor site. Age, sex, race and year of diagnosis were also included in the nomogram. The detailed scores for each characteristic are presented in eTable 7.

The calibration curves displayed high internal and external consistency with the actual observations for the survival probability of the training (C-index: 0.732) and testing sets (C-index: 0.748; Figures 8B–D). Meanwhile, for predicting 1-year survival, the AUCs of the training and testing sets were 0.782 and 0.796, respectively (Figure 8E). For predicting 3-year survival, the AUCs of the training and testing sets were 0.771 and 0.798, respectively (Figure 8F). For predicting 5-year survival, the AUCs of the training and testing sets were 0.777 and 0.810, respectively (Figure 8G).

## Discussion

In this large population-based study, the incidence of carcinosarcoma has continued to increase in the past decades, reaching a plateau from 2012 to 2019. This may reflect changes in medical practice, such as the more extensive use of cancer screening, biopsy, and better recognition by pathologists (5, 10, 24). The sex disparity in incidence has increased over time, mainly because of the dramatic increase in the female incidence rate. Carcinosarcoma mainly occurs in older patients, but its incidence continues to increase in younger patients. Previous studies demonstrated that black women have an increased risk of uterine carcinosarcoma, which may suggest a possible future demographic change in carcinosarcoma because the uterus is the dominant site of carcinosarcoma (10, 25, 26). Another possible explanation for the steep increase in incidence in the black population may be socioeconomic disparities (including low income, insurance status, health services) (25, 27, 28). With the rapid development of health inspection and imaging modalities, the incidence of patients diagnosed with unknown stage declined over the past decades. The most rapid increase in the incidence of localized stage tumors was accompanied by an increase in regional and distant stage tumors, which suggests the significance of prevention and early detection of carcinosarcoma. Mortality patterns reflect incidence trends and treatment effectiveness, with increased slowing for women, white population, and distant stage, and stabilizing rate for regional stage, while decreasing for ≥70 years old. The rapid decline in mortality among men may be due in part to reduction in smoking (29). Thus, while medical advances have slowed the trend of rising mortality, targeted cancer control treatments remain urgent (11, 25, 27, 28, 30). Consistent with the overall rising incidence and slowing death trend, the prevalence of carcinosarcoma has increased year-by-year, mainly in women, elderly, blacks, and localized stage. The prognosis of carcinosarcoma is far worse than that of most other solid tumors (29). The 5-year survival rate of carcinosarcoma was only 32.8%, similar to the previous studies of uterine and ovarian carcinosarcoma (29.8–37%) (5, 6, 23, 27, 31). Hence, effective prevention and treatment for carcinosarcoma are lacking and urgently needed.

In women, the uterus and ovary are the main sites of carcinosarcoma, which accounted for approximately 5% of all uterine cancers and 1–3% of all malignant ovarian tumors (3, 23, 31). In men, the lung and bronchus is the most common primary site of carcinosarcoma, followed by the urinary bladder and salivary gland. Although there have been some case reports and analyses of single-site carcinosarcoma, distribution and gender disparities of carcinosarcoma are new and comprehensive understanding of carcinosarcoma, facilitating disease screening and medical surveillance for future research (6, 7, 9, 32–34).

Despite the increasing interest of the medical community toward carcinosarcoma, there is still a scarcity of specific guidelines for its management (9, 27, 35). Surgery remains the predominant initial treatment for localized stage, whereas due to the high recurrence rate of carcinosarcoma, the proportion of combination therapy is more pronounced in regional and distant stages. Most studies support an improved survival benefit with combination therapy, but the incidence of carcinosarcoma is too low to provide prospective clinical trial support (22, 24, 36–41). Older patients prefer surgery alone rather than adjuvant chemotherapy or chemoradiotherapy after surgical resection. Meanwhile, a substantial proportion of patients were undertreated, especially older patients (≥70 years) with distant stage. Older patients with more comorbidities and worse performance scores limit aggressive treatment, and these differences may lead to poorer survival (41). However, these data should be interpreted with caution for most cancer types, as the SEER database provides only partial treatment information (chemotherapy, radiotherapy, and surgery) and does not include targeted therapy and immunotherapy (42). Of note, targeted therapy or immunotherapy was not available as a first-line option for carcinosarcoma to date, and thus would not affect the data analysis in this study (27, 43).

To further explore the risk factors for patients with carcinosarcoma, age, sex, race, year of diagnosis, disease stage, primary tumor site, and treatment correlated with OS. Older age, male sex, black, earlier year of diagnosis, advanced stage, and carcinosarcoma originating from the gallbladder and soft tissue including heart were associated with poorer prognosis. Identifying risk factors is emphasized to improve the outcome of carcinosarcoma. Explicating prognostic differences due to primary site will provide the basis for treatment and follow-up strategies. Survival benefits from all treatment modalities, especially triple therapies. This result demonstrates the importance of aggressive treatment in fighting carcinosarcoma invasion (2, 4, 12, 22, 33). A prognostic nomogram was constructed based on these seven risk factors. The total score was calculated using the quantitative score of each factor, and the 1-, 3-, and 5-year survival rate were scientifically and accurately predicted. Due to the inclusion of the primary tumor site, the predictive model is applicable to a wider population of patients and has a better predictive probability than other single-site predictive models (11, 30). In summary, this simple but effective model could be used to individualize prognostic assessment of carcinosarcoma and will facilitate clinical decision-making.

## Limitations and strengths

This study had several limitations. First, the analysis based on the SEER database was retrospective, and some information, such as specific chemotherapy drugs was lacking. Second, there was a lag in the data, with some variables censored after 2015. Finally, as definitive diagnosis of carcinosarcoma is difficult, the incidence and prevalence may be underestimated. Despite some limitations, this study offered the advantage of providing nationally representative epidemiological data, as well as survival data. To the best of our knowledge, this is the first, largest, and most up-to-date comprehensive study of carcinosarcoma that integrates multiple primary tumor sites, presenting a detailed analysis of treatment and prognosis. Therefore, this study provides significant and comprehensive information for studying carcinosarcoma.

## Conclusions

In this study, the incidence has continued to increase over the past decades, with increased acceleration in the localized stage, reflecting improved early detection. The increasing trend in mortality has slowed and declined rapidly among men; hence, the prevalence of carcinosarcoma has increased. Nonetheless, the survival of patients with carcinosarcoma remains poor, reflecting the urgency to improve early detection and explore targeted cancer control treatments. Explicating distribution and gender disparities of carcinosarcoma may facilitate disease screening and medical surveillance. All treatment modalities offer survival benefits, especially triple therapy. Differences in treatment patterns, comorbidities, and performance scores may explain the inferior prognosis of older patients. Furthermore, according to the risk factors in the multivariate analysis, a nomogram was constructed to predict the survival probability of carcinosarcoma, which demonstrated a good predictive capacity and could guide the surveillance, treatment, and follow-up strategies.

## Data availability statement

The original contributions presented in the study are included in the article/Supplementary material, further inquiries can be directed to the corresponding author/s.

## Ethics statement

Ethical review and approval was not required for the study on human participants in accordance with the local legislation and institutional requirements. Written informed consent to participate in this study was provided by the participants' legal guardian/next of kin. Written informed consent was obtained from the individual(s) for the publication of any potentially identifiable images or data included in this article.

## Author contributions

MC and WH: full access to all of the data in the study, take responsibility for the integrity of the data and the accuracy of the data analysis, concept and design, administrative, and technical or material support. MC, XH, QY, JZ, JP, DW, and KT: acquisition and analysis or interpretation of data. MC: drafting of the manuscript. MC, XH, and QY: statistical analysis. WH: supervision. All authors: critical revision of the manuscript for important intellectual content. All authors contributed to the article and approved the submitted version.

## Conflict of interest

The authors declare that the research was conducted in the absence of any commercial or financial relationships that could be construed as a potential conflict of interest.

## Publisher's note

All claims expressed in this article are solely those of the authors and do not necessarily represent those of their affiliated organizations, or those of the publisher, the editors and the reviewers. Any product that may be evaluated in this article, or claim that may be made by its manufacturer, is not guaranteed or endorsed by the publisher.

## Abbreviations

AAPC: average annual percent change
APC: annual percent change
AUC: area under the curve
CI: confidence interval
C-index: concordance indexes
HR: hazard ratio
ICD-O-3, International Classification of Diseases for Oncology: 3rd edition
OS: overall survival
ROC: receiver operating characteristic
SEER, Surveillance, Epidemiology: and End Results.
