# Supplementary material for: Epidemiology and prediction model of patients with carcinosarcoma in the United States
Source: Front Public Health. 2022 Nov 28;10:1038211. doi: 10.3389/fpubh.2022.1038211 (PMC9742429; doi:10.3389/fpubh.2022.1038211)
Supplement: Supplementary file 1 [file Data_Sheet_1.docx]

**Supplementary content**

**eTable1.** The age-adjusted incidence of carcinosarcoma by sex (1975–2019), age (1975–2019), race (1975–2019) and disease stage (1975–2015), SEER-8.

**eTable2.** Trends in the incidence rates of carcinosarcoma by sex (1975–2019), age (1975–2019), race (1975–2019) and disease stage (1975–2015), SEER-8.

**eTable3**. Twenty-year prevalence of carcinosarcoma, SEER-8.

**eTable4.** The age-adjusted mortality of carcinosarcoma by sex (1975–2019), age (1975–2019), race (1975–2019) and disease stage (1975–2015), SEER-8.

**eTable5.** Trends in the mortality rates of carcinosarcoma by sex (1975–2019), age (1975–2019), race (1975–2019) and disease stage (1975–2015), SEER-8.

**eTable6.** Univariate and multivariate regression analysis for carcinosarcoma, SEER-17, 2000–2019.

**eTable7.** Detailed nomogram score of every variable in the nomogram.

**eTable1. The age-adjusted incidence of carcinosarcoma by sex (1975–2019), age (1975–2019), race (1975–2019) and disease stage (1975–2015), SEER-8.**

| **Year** | **Sex** | | |  | **Age** | | | |  | **Race** | | |  | **Disease stage** | | | |
| --- | --- | --- | --- | --- | --- | --- | --- | --- | --- | --- | --- | --- | --- | --- | --- | --- | --- |
|  | Both sexes | Female | Male |  | ≤ 49 | 50–59 | 60–69 | ≥ 70 |  | White | Black | Other |  | Localized | Regional | Distant | Unstaged |
| 1975 | 0.46 | 0.83 | 0.07 |  | 0.03 | 1.29 | 1.63 | 2.74 |  | 0.44 | 0.99 | 0.18 |  | 0.20 | 0.09 | 0.08 | 0.10 |
| 1976 | 0.42 | 0.82 | 0.01 |  | 0.04 | 1.17 | 1.51 | 2.40 |  | 0.43 | 0.71 | 0.00 |  | 0.18 | 0.06 | 0.15 | 0.04 |
| 1977 | 0.45 | 0.81 | 0.09 |  | 0.05 | 0.58 | 2.02 | 3.06 |  | 0.44 | 0.60 | 0.49 |  | 0.14 | 0.09 | 0.15 | 0.08 |
| 1978 | 0.57 | 1.05 | 0.07 |  | 0.05 | 1.27 | 2.90 | 2.71 |  | 0.54 | 1.26 | 0.24 |  | 0.19 | 0.11 | 0.17 | 0.10 |
| 1979 | 0.42 | 0.74 | 0.08 |  | 0.03 | 0.52 | 2.23 | 2.46 |  | 0.42 | 0.49 | 0.23 |  | 0.17 | 0.07 | 0.12 | 0.05 |
| 1980 | 0.55 | 1.03 | 0.06 |  | 0.02 | 0.69 | 2.69 | 3.72 |  | 0.55 | 0.79 | 0.36 |  | 0.23 | 0.07 | 0.21 | 0.04 |
| 1981 | 0.57 | 1.07 | 0.05 |  | 0.04 | 0.75 | 2.57 | 3.77 |  | 0.61 | 0.46 | 0.21 |  | 0.17 | 0.07 | 0.24 | 0.09 |
| 1982 | 0.54 | 1.02 | 0.05 |  | 0.07 | 0.81 | 2.38 | 3.13 |  | 0.58 | 0.52 | 0.20 |  | 0.22 | 0.07 | 0.21 | 0.05 |
| 1983 | 0.52 | 0.95 | 0.07 |  | 0.02 | 0.47 | 2.77 | 3.26 |  | 0.55 | 0.43 | 0.25 |  | 0.19 | 0.08 | 0.19 | 0.07 |
| 1984 | 0.59 | 1.14 | 0.03 |  | 0.01 | 0.71 | 2.86 | 4.00 |  | 0.62 | 0.78 | 0.24 |  | 0.15 | 0.08 | 0.31 | 0.06 |
| 1985 | 0.53 | 1.02 | 0.03 |  | 0.05 | 1.26 | 2.09 | 2.89 |  | 0.53 | 0.83 | 0.35 |  | 0.14 | 0.06 | 0.25 | 0.09 |
| 1986 | 0.59 | 1.12 | 0.04 |  | 0.01 | 0.85 | 2.47 | 4.16 |  | 0.62 | 0.60 | 0.34 |  | 0.19 | 0.13 | 0.22 | 0.05 |
| 1987 | 0.54 | 0.97 | 0.09 |  | 0.06 | 0.73 | 1.86 | 3.71 |  | 0.56 | 0.72 | 0.17 |  | 0.19 | 0.10 | 0.19 | 0.06 |
| 1988 | 0.58 | 1.06 | 0.07 |  | 0.02 | 0.91 | 2.25 | 3.96 |  | 0.60 | 0.57 | 0.42 |  | 0.16 | 0.14 | 0.21 | 0.06 |
| 1989 | 0.64 | 1.19 | 0.06 |  | 0.05 | 0.84 | 2.64 | 4.07 |  | 0.67 | 0.87 | 0.20 |  | 0.20 | 0.12 | 0.27 | 0.05 |
| 1990 | 0.60 | 1.12 | 0.07 |  | 0.08 | 1.19 | 2.70 | 2.95 |  | 0.63 | 0.54 | 0.45 |  | 0.18 | 0.10 | 0.29 | 0.04 |
| 1991 | 0.57 | 1.09 | 0.04 |  | 0.07 | 1.05 | 1.85 | 3.60 |  | 0.61 | 0.47 | 0.38 |  | 0.17 | 0.14 | 0.21 | 0.06 |
| 1992 | 0.49 | 0.89 | 0.09 |  | 0.04 | 0.74 | 1.79 | 3.32 |  | 0.50 | 0.68 | 0.28 |  | 0.18 | 0.07 | 0.22 | 0.03 |
| 1993 | 0.58 | 1.08 | 0.07 |  | 0.06 | 0.65 | 2.01 | 4.06 |  | 0.61 | 0.66 | 0.35 |  | 0.14 | 0.16 | 0.26 | 0.03 |
| 1994 | 0.62 | 1.11 | 0.13 |  | 0.06 | 0.68 | 2.70 | 3.97 |  | 0.68 | 0.64 | 0.21 |  | 0.24 | 0.12 | 0.22 | 0.05 |
| 1995 | 0.63 | 1.18 | 0.07 |  | 0.06 | 0.91 | 2.50 | 3.88 |  | 0.64 | 1.04 | 0.20 |  | 0.15 | 0.17 | 0.28 | 0.03 |
| 1996 | 0.71 | 1.29 | 0.11 |  | 0.05 | 1.47 | 2.30 | 4.48 |  | 0.76 | 0.66 | 0.40 |  | 0.24 | 0.20 | 0.21 | 0.06 |
| 1997 | 0.47 | 0.85 | 0.08 |  | 0.04 | 0.64 | 1.83 | 2.99 |  | 0.47 | 0.69 | 0.31 |  | 0.14 | 0.12 | 0.17 | 0.04 |
| 1998 | 0.46 | 0.83 | 0.07 |  | 0.05 | 0.61 | 2.23 | 2.52 |  | 0.49 | 0.38 | 0.34 |  | 0.14 | 0.12 | 0.17 | 0.03 |
| 1999 | 0.62 | 1.11 | 0.12 |  | 0.05 | 0.95 | 1.94 | 4.12 |  | 0.64 | 0.74 | 0.40 |  | 0.21 | 0.14 | 0.23 | 0.03 |
| 2000 | 0.57 | 1.03 | 0.10 |  | 0.13 | 0.59 | 2.13 | 3.18 |  | 0.62 | 0.54 | 0.28 |  | 0.18 | 0.15 | 0.23 | 0.02 |
| 2001 | 0.60 | 1.11 | 0.09 |  | 0.06 | 1.06 | 1.57 | 3.93 |  | 0.68 | 0.35 | 0.34 |  | 0.17 | 0.20 | 0.20 | 0.03 |
| 2002 | 0.65 | 1.18 | 0.10 |  | 0.06 | 0.87 | 3.51 | 3.14 |  | 0.63 | 1.04 | 0.47 |  | 0.17 | 0.19 | 0.27 | 0.02 |
| 2003 | 0.62 | 1.15 | 0.07 |  | 0.06 | 1.20 | 2.08 | 3.32 |  | 0.64 | 0.81 | 0.36 |  | 0.18 | 0.18 | 0.23 | 0.01 |
| 2004 | 0.64 | 1.23 | 0.04 |  | 0.05 | 0.79 | 3.06 | 3.41 |  | 0.70 | 0.55 | 0.35 |  | 0.20 | 0.15 | 0.26 | 0.03 |
| 2005 | 0.65 | 1.24 | 0.04 |  | 0.10 | 1.15 | 2.38 | 3.02 |  | 0.68 | 0.79 | 0.34 |  | 0.14 | 0.21 | 0.27 | 0.02 |
| 2006 | 0.64 | 1.23 | 0.04 |  | 0.05 | 1.21 | 2.46 | 3.09 |  | 0.64 | 1.08 | 0.33 |  | 0.17 | 0.17 | 0.27 | 0.03 |
| 2007 | 0.59 | 1.12 | 0.04 |  | 0.05 | 0.84 | 2.14 | 3.21 |  | 0.60 | 0.90 | 0.29 |  | 0.14 | 0.19 | 0.23 | 0.02 |
| 2008 | 0.75 | 1.45 | 0.04 |  | 0.09 | 1.07 | 2.75 | 3.76 |  | 0.75 | 1.07 | 0.43 |  | 0.23 | 0.17 | 0.34 | 0.01 |
| 2009 | 0.72 | 1.40 | 0.02 |  | 0.06 | 1.32 | 2.38 | 3.51 |  | 0.69 | 1.31 | 0.44 |  | 0.22 | 0.20 | 0.27 | 0.02 |
| 2010 | 0.76 | 1.46 | 0.04 |  | 0.08 | 0.94 | 2.95 | 3.64 |  | 0.77 | 0.88 | 0.59 |  | 0.24 | 0.19 | 0.29 | 0.04 |
| 2011 | 0.80 | 1.52 | 0.05 |  | 0.09 | 1.07 | 3.07 | 3.52 |  | 0.79 | 1.22 | 0.50 |  | 0.23 | 0.20 | 0.34 | 0.02 |
| 2012 | 0.90 | 1.70 | 0.08 |  | 0.09 | 1.43 | 3.06 | 4.03 |  | 0.88 | 1.45 | 0.56 |  | 0.24 | 0.29 | 0.34 | 0.04 |
| 2013 | 0.87 | 1.58 | 0.14 |  | 0.10 | 1.19 | 3.11 | 3.68 |  | 0.93 | 0.97 | 0.48 |  | 0.26 | 0.26 | 0.32 | 0.02 |
| 2014 | 0.91 | 1.73 | 0.06 |  | 0.10 | 1.32 | 3.12 | 3.74 |  | 0.84 | 1.91 | 0.46 |  | 0.23 | 0.29 | 0.36 | 0.02 |
| 2015 | 0.92 | 1.74 | 0.08 |  | 0.06 | 1.29 | 3.33 | 3.89 |  | 0.88 | 1.50 | 0.64 |  | 0.26 | 0.25 | 0.38 | 0.03 |
| 2016 | 0.85 | 1.61 | 0.08 |  | 0.06 | 1.25 | 3.01 | 3.46 |  | 0.81 | 1.47 | 0.58 |  | - | - | - | - |
| 2017 | 0.89 | 1.76 | 0.00 |  | 0.07 | 1.20 | 3.04 | 3.68 |  | 0.79 | 1.57 | 0.82 |  | - | - | - | - |
| 2018 | 0.80 | 1.58 | 0.01 |  | 0.05 | 0.90 | 2.93 | 3.35 |  | 0.80 | 1.33 | 0.40 |  | - | - | - | - |
| 2019 | 0.91 | 1.73 | 0.07 |  | 0.06 | 1.31 | 3.25 | 3.33 |  | 0.89 | 1.40 | 0.59 |  | - | - | - | - |

**eTable2. Trends in incidence rates of carcinosarcoma by sex (1975–2019), age (1975–2019), race (1975–2019) and disease stage (1975–2015)^a^, SEER-8.**

|  | **Subgroups** | **Trend 1** | | **Trend 2** | | **Trend 3** | | **AAPC** | | | | | |
| --- | --- | --- | --- | --- | --- | --- | --- | --- | --- | --- | --- | --- | --- |
|  |  | Years | APC | Years | APC | Years | APC | 1975–2014**^a^** | P | 2015–2019**^a^** | P | 1975–2019**^a^** | P |
| **Sex** | Both sexes | 1975–2007 | 0.8^b^ | 2007–2012 | 6.5 | 2012–2019 | -0.2 | 1.4^b^ | 0.002 | -0.2 | 0.862 | 1.3^b^ | 0.006 |
|  | Female | 1975–2000 | 0.6^b^ | 2000–2019 | 2.7^b^ |  |  | 1.4^b^ | <0.001 | 2.7^b^ | <0.001 | 1.5^b^ | <0.001 |
|  | Male | 1975–2019 | 0.0 |  |  |  |  | 0.0 | 0.986 | 0.0 | 0.986 | 0.0 | 0.986 |
| **Age**  **(years)** | ≤ 49 | 1975–2019 | 1.6^b^ |  |  |  |  | 1.6^b^ | 0.001 | 1.6^b^ | 0.001 | 1.6^b^ | 0.001 |
|  | 50–59 | 1975–2019 | 0.9^b^ |  |  |  |  | 0.9^b^ | 0.003 | 0.9^b^ | 0.003 | 0.9^b^ | 0.003 |
|  | 60–69 | 1975–1978 | 21.8 | 1978–1997 | -0.9 | 1997–2019 | 1.9^b^ | 1.9 | 0.109 | 1.9^b^ | <0.001 | 1.9 | 0.073 |
|  | ≥70 | 1975–1984 | 3.9 | 1984–2019 | -0.1 |  |  | 0.8 | 0.128 | -0.1 | 0.448 | 0.7 | 0.143 |
| **Race** | White | 1975–2019 | 1.3^b^ |  |  |  |  | 1.3^b^ | <0.001 | 1.3^b^ | <0.001 | 1.3^b^ | <0.001 |
|  | Black | 1975–1998 | -0.8 | 1998–2019 | 4.7^b^ |  |  | 1.4^b^ | 0.003 | 4.7^b^ | <0.001 | 1.8^b^ | 0.005 |
|  | Other | 1975–2019 | 2.5^b^ |  |  |  |  | 2.5^b^ | <0.001 | 2.5^b^ | <0.001 | 2.5^b^ | <0.001 |
| **Disease**  **stage^a^** | Localized | 1975–2005 | -0.2 | 2005–2015 | 4.2^b^ |  |  | 0.4 | 0.225 | 4.2^b^ | 0.008 | 0.9^b^ | 0.045 |
|  | Regional | 1975–2015 | 3.3^b^ |  |  |  |  | 3.3^b^ | <0.001 | 3.3^b^ | <0.001 | 3.3^b^ | <0.001 |
|  | Distant | 1975–1984 | 9.5^b^ | 1984–1998 | -1.7 | 1998–2015 | 3.4^b^ | 2.8^b^ | <0.001 | 3.4^b^ | <0.001 | 2.9^b^ | <0.001 |
|  | Unstaged | 1975–2015 | -3.3^b^ |  |  |  |  | -3.3^b^ | <0.001 | -3.3^b^ | <0.001 | -3.3^b^ | <0.001 |

^a^ Disease stage was referenced to SEER historic stage A as of 2015, so the time periods for AAPC were 1975–2010, 2011–2015 and 1975–2015, respectively.

^b^ The APC or AAPC is significantly different from zero (P < 0.05).

SEER, Surveillance, Epidemiology, and End Results; AAPC, average annual percent change; APC, annual percent change based on incidence rates adjusted to the 2000 US standard population.

**eTable3. Twenty-year prevalence of carcinosarcoma, SEER-8.**

| **Year** | **Sex** | | |  | **Age** | | | |  | **Race** | | |  | **Disease stage** | | | |
| --- | --- | --- | --- | --- | --- | --- | --- | --- | --- | --- | --- | --- | --- | --- | --- | --- | --- |
|  | Both sexes | Female | Male |  | ≤ 49 | 50–59 | 60–69 | ≥ 70 |  | White | Black | Other |  | Localized | Regional | Distant | Unstaged |
| 1996 | - | - | - |  | - | - | - | - |  | - | - | - |  | 0.24 | 0.18 | 0.16 | 0.03 |
| 1997 | - | - | - |  | - | - | - | - |  | - | - | - |  | 0.34 | 0.21 | 0.18 | 0.03 |
| 1998 | - | - | - |  | - | - | - | - |  | - | - | - |  | 0.40 | 0.27 | 0.18 | 0.03 |
| 1999 | 0.47 | 0.79 | 0.09 |  | 0.03 | 0.08 | 0.11 | 0.24 |  | 0.45 | 1.05 | 0.39 |  | 0.51 | 0.32 | 0.27 | 0.04 |
| 2000 | 0.72 | 1.22 | 0.12 |  | 0.11 | 0.12 | 0.20 | 0.30 |  | 0.72 | 1.34 | 0.48 |  | 0.56 | 0.34 | 0.29 | 0.04 |
| 2001 | 1.01 | 1.72 | 0.15 |  | 0.14 | 0.17 | 0.25 | 0.44 |  | 1.04 | 1.15 | 0.65 |  | 0.66 | 0.42 | 0.33 | 0.05 |
| 2002 | 1.16 | 2.04 | 0.14 |  | 0.15 | 0.21 | 0.37 | 0.43 |  | 1.14 | 2.04 | 0.81 |  | 0.69 | 0.44 | 0.41 | 0.04 |
| 2003 | 1.33 | 2.36 | 0.12 |  | 0.18 | 0.26 | 0.38 | 0.51 |  | 1.34 | 2.51 | 0.71 |  | 0.78 | 0.50 | 0.40 | 0.05 |
| 2004 | 1.48 | 2.64 | 0.11 |  | 0.19 | 0.29 | 0.47 | 0.53 |  | 1.52 | 2.18 | 0.85 |  | 0.85 | 0.50 | 0.45 | 0.04 |
| 2005 | 1.60 | 2.90 | 0.09 |  | 0.23 | 0.33 | 0.51 | 0.53 |  | 1.64 | 2.48 | 0.89 |  | 0.84 | 0.53 | 0.51 | 0.05 |
| 2006 | 1.70 | 3.08 | 0.10 |  | 0.22 | 0.37 | 0.58 | 0.54 |  | 1.72 | 2.67 | 1.06 |  | 0.90 | 0.55 | 0.50 | 0.06 |
| 2007 | 1.77 | 3.22 | 0.08 |  | 0.23 | 0.38 | 0.59 | 0.57 |  | 1.75 | 3.21 | 1.08 |  | 0.93 | 0.59 | 0.47 | 0.06 |
| 2008 | 1.97 | 3.59 | 0.09 |  | 0.27 | 0.41 | 0.64 | 0.65 |  | 1.94 | 3.49 | 1.17 |  | 1.04 | 0.62 | 0.54 | 0.04 |
| 2009 | 2.06 | 3.77 | 0.09 |  | 0.29 | 0.46 | 0.69 | 0.62 |  | 2.00 | 3.76 | 1.38 |  | 1.07 | 0.67 | 0.52 | 0.04 |
| 2010 | 2.18 | 4.00 | 0.10 |  | 0.30 | 0.49 | 0.72 | 0.68 |  | 2.15 | 3.78 | 1.44 |  | 1.15 | 0.66 | 0.57 | 0.05 |
| 2011 | 2.40 | 4.41 | 0.09 |  | 0.30 | 0.52 | 0.81 | 0.76 |  | 2.35 | 4.28 | 1.59 |  | 1.24 | 0.71 | 0.62 | 0.05 |
| 2012 | 2.61 | 4.78 | 0.15 |  | 0.34 | 0.58 | 0.89 | 0.80 |  | 2.57 | 4.40 | 1.75 |  | 1.31 | 0.78 | 0.67 | 0.06 |
| 2013 | 2.76 | 5.03 | 0.17 |  | 0.36 | 0.59 | 0.98 | 0.84 |  | 2.76 | 4.40 | 1.75 |  | 1.39 | 0.84 | 0.66 | 0.06 |
| 2014 | 2.94 | 5.42 | 0.11 |  | 0.40 | 0.64 | 1.04 | 0.86 |  | 2.86 | 5.27 | 1.88 |  | 1.44 | 0.91 | 0.70 | 0.06 |
| 2015 | 3.12 | 5.72 | 0.16 |  | 0.40 | 0.72 | 1.10 | 0.91 |  | 3.03 | 5.48 | 2.10 |  | 1.53 | 0.92 | 0.75 | 0.08 |
| 2016 | 3.21 | 5.87 | 0.18 |  | 0.40 | 0.77 | 1.15 | 0.89 |  | 3.11 | 5.38 | 2.20 |  | - | - | - | - |
| 2017 | 3.33 | 6.13 | 0.13 |  | 0.41 | 0.77 | 1.21 | 0.94 |  | 3.18 | 5.69 | 2.47 |  | - | - | - | - |
| 2018 | 3.36 | 6.20 | 0.10 |  | 0.40 | 0.78 | 1.25 | 0.92 |  | 3.21 | 6.26 | 2.27 |  | - | - | - | - |

^a^ Disease stage was referenced to SEER historic stage A as of 2015, so the time periods was 1996–2015.

**eTable4. The age-adjusted mortality of carcinosarcoma by sex (1975–2019), age (1975–2019), race (1975–2019) and disease stage (1975–2015), SEER-8.**

| **Year** | **Sex** | | |  | **Age** | | | |  | **Race** | | |  | **Disease stage** | | | |
| --- | --- | --- | --- | --- | --- | --- | --- | --- | --- | --- | --- | --- | --- | --- | --- | --- | --- |
|  | Both sexes | Female | Male |  | ≤ 49 | 50–59 | 60–69 | ≥ 70 |  | White | Black | Other |  | Localized | Regional | Distant | Unstaged |
| 1975 | 0.16 | 0.24 | 0.07 |  | 0.02 | 0.04 | 0.01 | 0.10 |  | 0.14 | 0.55 | 0.20 |  | 0.03 | 0.04 | 0.05 | 0.05 |
| 1976 | 0.26 | 0.44 | 0.01 |  | 0.01 | 0.05 | 0.05 | 0.15 |  | 0.24 | 1.06 | 0.00 |  | 0.07 | 0.05 | 0.11 | 0.04 |
| 1977 | 0.42 | 0.66 | 0.09 |  | 0.02 | 0.07 | 0.10 | 0.23 |  | 0.42 | 0.65 | 0.20 |  | 0.12 | 0.06 | 0.15 | 0.08 |
| 1978 | 0.34 | 0.55 | 0.06 |  | 0.00 | 0.05 | 0.13 | 0.16 |  | 0.34 | 0.62 | 0.23 |  | 0.09 | 0.06 | 0.15 | 0.05 |
| 1979 | 0.44 | 0.69 | 0.09 |  | 0.02 | 0.08 | 0.11 | 0.24 |  | 0.40 | 1.70 | 0.18 |  | 0.09 | 0.10 | 0.18 | 0.08 |
| 1980 | 0.41 | 0.63 | 0.10 |  | 0.02 | 0.08 | 0.10 | 0.22 |  | 0.37 | 1.25 | 0.29 |  | 0.14 | 0.07 | 0.16 | 0.05 |
| 1981 | 0.51 | 0.84 | 0.02 |  | 0.01 | 0.07 | 0.16 | 0.28 |  | 0.50 | 0.99 | 0.32 |  | 0.18 | 0.08 | 0.20 | 0.05 |
| 1982 | 0.54 | 0.84 | 0.10 |  | 0.04 | 0.04 | 0.14 | 0.32 |  | 0.55 | 0.98 | 0.08 |  | 0.15 | 0.07 | 0.26 | 0.05 |
| 1983 | 0.45 | 0.75 | 0.03 |  | 0.01 | 0.08 | 0.14 | 0.21 |  | 0.45 | 0.71 | 0.27 |  | 0.13 | 0.08 | 0.18 | 0.06 |
| 1984 | 0.57 | 0.94 | 0.05 |  | 0.01 | 0.05 | 0.16 | 0.35 |  | 0.55 | 1.27 | 0.38 |  | 0.15 | 0.08 | 0.26 | 0.08 |
| 1985 | 0.51 | 0.82 | 0.07 |  | 0.01 | 0.05 | 0.17 | 0.29 |  | 0.49 | 1.18 | 0.28 |  | 0.13 | 0.05 | 0.24 | 0.09 |
| 1986 | 0.49 | 0.79 | 0.05 |  | 0.02 | 0.07 | 0.15 | 0.25 |  | 0.50 | 0.75 | 0.25 |  | 0.10 | 0.07 | 0.27 | 0.04 |
| 1987 | 0.50 | 0.82 | 0.05 |  | 0.02 | 0.08 | 0.13 | 0.26 |  | 0.47 | 1.07 | 0.44 |  | 0.14 | 0.07 | 0.24 | 0.05 |
| 1988 | 0.55 | 0.87 | 0.07 |  | 0.02 | 0.05 | 0.15 | 0.33 |  | 0.56 | 0.79 | 0.21 |  | 0.17 | 0.10 | 0.21 | 0.07 |
| 1989 | 0.58 | 0.91 | 0.10 |  | 0.03 | 0.06 | 0.18 | 0.31 |  | 0.56 | 1.55 | 0.21 |  | 0.19 | 0.09 | 0.21 | 0.09 |
| 1990 | 0.56 | 0.96 | 0.06 |  | 0.04 | 0.10 | 0.20 | 0.23 |  | 0.53 | 1.33 | 0.63 |  | 0.14 | 0.10 | 0.27 | 0.05 |
| 1991 | 0.61 | 0.97 | 0.10 |  | 0.04 | 0.07 | 0.12 | 0.38 |  | 0.61 | 1.26 | 0.18 |  | 0.15 | 0.11 | 0.26 | 0.08 |
| 1992 | 0.41 | 0.67 | 0.05 |  | 0.02 | 0.06 | 0.10 | 0.23 |  | 0.39 | 0.94 | 0.32 |  | 0.10 | 0.09 | 0.19 | 0.04 |
| 1993 | 0.53 | 0.84 | 0.06 |  | 0.04 | 0.04 | 0.13 | 0.32 |  | 0.49 | 1.50 | 0.17 |  | 0.11 | 0.12 | 0.27 | 0.03 |
| 1994 | 0.52 | 0.81 | 0.09 |  | 0.03 | 0.05 | 0.13 | 0.31 |  | 0.52 | 0.93 | 0.26 |  | 0.13 | 0.10 | 0.23 | 0.05 |
| 1995 | 0.53 | 0.82 | 0.10 |  | 0.01 | 0.07 | 0.16 | 0.28 |  | 0.47 | 1.56 | 0.33 |  | 0.13 | 0.13 | 0.22 | 0.04 |
| 1996 | 0.57 | 0.90 | 0.14 |  | 0.03 | 0.11 | 0.14 | 0.29 |  | 0.58 | 1.05 | 0.27 |  | 0.11 | 0.17 | 0.26 | 0.04 |
| 1997 | 0.64 | 1.01 | 0.13 |  | 0.01 | 0.09 | 0.18 | 0.36 |  | 0.60 | 1.52 | 0.47 |  | 0.17 | 0.17 | 0.24 | 0.05 |
| 1998 | 0.58 | 0.92 | 0.13 |  | 0.03 | 0.05 | 0.18 | 0.33 |  | 0.59 | 0.64 | 0.43 |  | 0.19 | 0.12 | 0.20 | 0.06 |
| 1999 | 0.45 | 0.70 | 0.10 |  | 0.01 | 0.06 | 0.17 | 0.21 |  | 0.43 | 1.03 | 0.29 |  | 0.16 | 0.11 | 0.16 | 0.01 |
| 2000 | 0.59 | 0.93 | 0.10 |  | 0.02 | 0.08 | 0.13 | 0.35 |  | 0.55 | 1.58 | 0.34 |  | 0.15 | 0.15 | 0.24 | 0.04 |
| 2001 | 0.48 | 0.74 | 0.11 |  | 0.02 | 0.08 | 0.08 | 0.30 |  | 0.49 | 0.84 | 0.23 |  | 0.12 | 0.16 | 0.18 | 0.02 |
| 2002 | 0.59 | 0.93 | 0.13 |  | 0.03 | 0.07 | 0.18 | 0.30 |  | 0.58 | 0.96 | 0.40 |  | 0.14 | 0.21 | 0.20 | 0.02 |
| 2003 | 0.52 | 0.86 | 0.07 |  | 0.02 | 0.09 | 0.16 | 0.26 |  | 0.47 | 1.18 | 0.48 |  | 0.15 | 0.14 | 0.22 | 0.01 |
| 2004 | 0.56 | 0.93 | 0.08 |  | 0.03 | 0.05 | 0.16 | 0.33 |  | 0.54 | 1.45 | 0.29 |  | 0.16 | 0.15 | 0.22 | 0.03 |
| 2005 | 0.57 | 0.95 | 0.07 |  | 0.03 | 0.10 | 0.15 | 0.29 |  | 0.58 | 0.78 | 0.34 |  | 0.17 | 0.17 | 0.21 | 0.02 |
| 2006 | 0.55 | 0.93 | 0.07 |  | 0.04 | 0.10 | 0.13 | 0.28 |  | 0.54 | 1.51 | 0.14 |  | 0.09 | 0.14 | 0.28 | 0.03 |
| 2007 | 0.54 | 0.90 | 0.07 |  | 0.03 | 0.07 | 0.17 | 0.26 |  | 0.54 | 0.81 | 0.36 |  | 0.11 | 0.14 | 0.27 | 0.02 |
| 2008 | 0.54 | 0.93 | 0.03 |  | 0.03 | 0.09 | 0.18 | 0.24 |  | 0.49 | 1.42 | 0.36 |  | 0.08 | 0.13 | 0.29 | 0.04 |
| 2009 | 0.58 | 1.03 | 0.01 |  | 0.03 | 0.08 | 0.15 | 0.32 |  | 0.53 | 1.76 | 0.24 |  | 0.17 | 0.12 | 0.27 | 0.01 |
| 2010 | 0.62 | 1.06 | 0.06 |  | 0.05 | 0.06 | 0.23 | 0.28 |  | 0.56 | 1.39 | 0.51 |  | 0.16 | 0.19 | 0.23 | 0.03 |
| 2011 | 0.55 | 0.95 | 0.04 |  | 0.05 | 0.09 | 0.15 | 0.26 |  | 0.51 | 1.23 | 0.41 |  | 0.15 | 0.13 | 0.26 | 0.02 |
| 2012 | 0.59 | 1.03 | 0.03 |  | 0.02 | 0.10 | 0.17 | 0.30 |  | 0.54 | 1.55 | 0.37 |  | 0.16 | 0.15 | 0.26 | 0.02 |
| 2013 | 0.60 | 1.00 | 0.10 |  | 0.05 | 0.11 | 0.16 | 0.28 |  | 0.56 | 1.38 | 0.40 |  | 0.13 | 0.16 | 0.29 | 0.01 |
| 2014 | 0.55 | 0.91 | 0.11 |  | 0.04 | 0.07 | 0.18 | 0.26 |  | 0.52 | 1.41 | 0.29 |  | 0.12 | 0.16 | 0.25 | 0.03 |
| 2015 | 0.56 | 0.99 | 0.04 |  | 0.04 | 0.05 | 0.19 | 0.29 |  | 0.51 | 1.58 | 0.28 |  | 0.10 | 0.17 | 0.28 | 0.02 |
| 2016 | 0.60 | 1.06 | 0.04 |  | 0.04 | 0.07 | 0.17 | 0.32 |  | 0.55 | 1.39 | 0.44 |  | - | - | - | - |
| 2017 | 0.60 | 1.04 | 0.05 |  | 0.04 | 0.10 | 0.16 | 0.30 |  | 0.56 | 1.19 | 0.43 |  | - | - | - | - |
| 2018 | 0.52 | 0.93 | 0.04 |  | 0.03 | 0.06 | 0.17 | 0.27 |  | 0.50 | 0.94 | 0.42 |  | - | - | - | - |
| 2019 | 0.51 | 0.90 | 0.03 |  | 0.04 | 0.07 | 0.17 | 0.22 |  | 0.49 | 0.78 | 0.35 |  | - | - | - | - |

**eTable5. Trends in mortality rates of carcinosarcoma by sex (1975–2019), age (1975–2019), race (1975–2019) and disease stage (1975–2015)^a^, SEER-8.**

|  | **Subgroups** | **Trend 1** | | **Trend 2** | | **Trend 3** | | **AAPC** | | | | | |
| --- | --- | --- | --- | --- | --- | --- | --- | --- | --- | --- | --- | --- | --- |
|  |  | Years | APC | Years | APC | Years | APC | 1975–2014^a^ | P | 2015–2019^a^ | P | 1975–2019^a^ | P |
| **Sex** | Both sexes | 1975–1981 | 13.5^b^ | 1981–2019 | 0.2 |  |  | 2.2^b^ | <0.001 | 0.2 | 0.102 | 1.9^b^ | <0.001 |
|  | Female | 1975–1981 | 13.0^b^ | 1981–2019 | 0.5^b^ |  |  | 2.3^b^ | <0.001 | 0.5^b^ | 0.001 | 2.1^b^ | <0.001 |
|  | Male | 1975–1998 | 3.3^b^ | 1998–2019 | -4.1^b^ |  |  | 0.2 | 0.849 | -4.1^b^ | 0.003 | -0.3 | 0.743 |
| **Age (years)** | ≤ 49 | 1975–2019 | 2.0^b^ |  |  |  |  | 2.0^b^ | <0.001 | 2.0^b^ | <0.001 | 2.0^b^ | <0.001 |
|  | 50–59 | 1975–2019 | 0.8^b^ |  |  |  |  | 0.8^b^ | 0.013 | 0.8^b^ | 0.013 | 0.8^b^ | 0.013 |
|  | 60–69 | 1975–1977 | 192.1 | 1977–2019 | 0.6^b^ |  |  | 6.3 | 0.125 | 0.6^b^ | 0.003 | 5.6 | 0.120 |
|  | ≥ 70 | 1975–1982 | 12.3^b^ | 1982–2019 | -0.3 |  |  | 1.9^b^ | 0.019 | -0.3 | 0.221 | 1.6^b^ | 0.022 |
| **Race** | White | 1975–1977 | 55.4 | 1977–1982 | 7.8 | 1982–2019 | 0.1 | 3.3^b^ | 0.037 | 0.1 | 0.587 | 3.0^b^ | 0.036 |
|  | Black | 1975–2016 | 1.0^b^ | 2016–2019 | -19.0 |  |  | 1.0^b^ | 0.007 | -14.4 | 0.077 | -0.5 | 0.524 |
|  | Other | 1975–2019 | 1.3^b^ |  |  |  |  | 1.3^b^ | 0.029 | 1.3^b^ | 0.029 | 1.3^b^ | 0.029 |
| **Disease stage^a^** | Localized | 1975–1981 | 17.2 | 1981–2015 | -0.4 |  |  | 2.4 | 0.113 | -0.4 | 0.324 | 2.1 | 0.124 |
|  | Regional | 1975–1997 | 5.0^b^ | 1997–2015 | 0.4 |  |  | 3.2^b^ | <0.001 | 0.4 | 0.582 | 2.9^b^ | <0.001 |
|  | Distant | 1975–1982 | 15.5^b^ | 1982–2001 | -0.7 | 2001–2015 | 1.9^b^ | 3.0^b^ | 0.001 | 1.9^b^ | 0.015 | 2.9^b^ | <0.001 |
|  | Unstaged | 1975–2015 | -3.4^b^ |  |  |  |  | -3.4^b^ | <0.001 | -3.4^b^ | <0.001 | -3.4^b^ | <0.001 |

^a^ Disease stage was referenced to SEER historic stage A as of 2015, so the time periods for AAPC were 1975–2010, 2011–2015 and 1975–2015, respectively.

^b^ The APC or AAPC is significantly different from zero (P < 0.05).

SEER, Surveillance, Epidemiology, and End Results; AAPC, average annual percent change; APC, annual percent change based on incidence rates adjusted to the 2000 US standard population.

**eTable6. Univariate and multivariate regression analysis for carcinosarcoma, SEER-17, 2000–2019.**

| **Characteristic** | **Univariate analysis** | | **Multivariate analysis** | |
| --- | --- | --- | --- | --- |
|  | **HR (95%CI)** | **P** | **HR (95%CI)** | **P** |
| **Age (year)** |  |  |  |  |
| ≤ 49 | 1 (Reference) | - | 1 (Reference) | - |
| 50–59 | 1.20 (1.10–1.40) | <0.001 | 1.34 (1.19–1.49) | <0.001 |
| 60–69 | 1.40 (1.30–1.60) | <0.001 | 1.49 (1.34–1.65) | <0.001 |
| ≥ 70 | 2.00 (1.80–2.30) | <0.001 | 1.86 (1.68–2.07) | <0.001 |
| **Sex** |  |  |  |  |
| Male | 1 (Reference) | - | 1 (Reference) | - |
| Female | 0.59 (0.52–0.67) | <0.001 | 0.70 (0.58–0.85) | <0.001 |
| **Race** |  |  |  |  |
| White | 1 (Reference) | - | 1 (Reference) | - |
| Black | 1.20 (1.11–1.24) | <0.001 | 1.17 (1.10–1.24) | <0.001 |
| Other | 0.90 (0.82–0.98) | 0.020 | 1.00 (0.91–1.09) | 0.953 |
| **Year of diagnosis** |  |  |  |  |
| 2000-2004 | 1 (Reference) | - | 1 (Reference) | - |
| 2005-2009 | 0.87 (0.81–0.93) | <0.001 | 0.90 (0.84–0.96) | <0.001 |
| 2010-2014 | 0.81 (0.76–0.86) | <0.001 | 0.88 (0.83–0.94) | <0.001 |
| 2015-2019 | 0.73 (0.68–0.78) | <0.001 | 0.87 (0.81–0.93) | <0.001 |
| **Disease stage** |  |  |  |  |
| Localized | 1 (Reference) | - | 1 (Reference) | - |
| Regional | 2.10 (2.00–2.30) | <0.001 | 2.41 (2.26–2.58) | <0.001 |
| Distant | 3.80 (3.60–4.00) | <0.001 | 4.57 (4.26–4.90) | <0.001 |
| **Primary tumor site** |  |  |  |  |
| Uterus | 1 (Reference) | - | 1 (Reference) | - |
| Ovary | 1.29 (1.22–1.37) | <0.001 | 0.78 (0.73–0.83) | <0.001 |
| Lung and bronchus | 2.02 (1.77–2.29) | <0.001 | 1.00 (0.84–1.18) | 0.959 |
| Breast | 0.58 (0.48–0.71) | <0.001 | 0.92 (0.75–1.13) | 0.434 |
| Urinary bladder | 1.63 (1.36–1.96) | <0.001 | 0.94 (0.74–1.18) | 0.590 |
| Peritoneum, omentum and mesentery | 1.48 (1.23–1.79) | <0.001 | 0.90 (0.75–1.09) | 0.297 |
| Soft tissue including heart | 1.67 (1.25–2.22) | <0.001 | 1.45 (1.09–1.94) | 0.012 |
| Salivary gland | 0.69 (0.46–1.04) | 0.073 | 0.56 (0.37–0.85) | 0.007 |
| Other non-epithelial skin | 0.75 (0.50–1.12) | 0.162 | 0.52 (0.34–0.80) | 0.003 |
| Gallbladder | 2.05 (1.45–2.90) | <0.001 | 1.57 (1.09–2.27) | 0.016 |
| **Treatment** |  |  |  |  |
| No treatment/Unknown | 1 (Reference) | - | 1 (Reference) | - |
| Single treatment, chemotherapy | 0.39 (0.34–0.44) | <0.001 | 0.31 (0.27–0.35) | <0.001 |
| Single treatment, radiotherapy | 0.64 (0.54–0.76) | <0.001 | 0.61 (0.52–0.72) | <0.001 |
| Single treatment, surgery | 0.20 (0.19–0.23) | <0.001 | 0.31 (0.28–0.34) | <0.001 |
| Chemotherapy+radiotherapy | 0.36 (0.30–0.44) | <0.001 | 0.32 (0.26–0.38) | <0.001 |
| Chemotherapy+surgery | 0.16 (0.15–0.18) | <0.001 | 0.17 (0.15–0.19) | <0.001 |
| Radiotherapy+surgery | 0.13 (0.12–0.15) | <0.001 | 0.22 (0.19–0.24) | <0.001 |
| Chemotherapy+radiotherapy  +surgery | 0.09 (0.08–0.10) | <0.001 | 0.13 (0.11–0.14) | <0.001 |

**eTable7. Detailed nomogram score of every variable in the nomogram.**

| **Predictive factors** | | |
| --- | --- | --- |
| **Variable** | **Category** | **Score** |
| **Age (year)** | ≤ 49 | 0 |
|  | 50–59 | 14 |
|  | 60–69 | 19 |
|  | ≥ 70 | 30 |
| **Sex** | Female | 0 |
|  | Male | 17 |
| **Race** | White | 0 |
|  | Black | 8 |
|  | Other | 0 |
| **Year of diagnosis** | 2000-2004 | 7 |
|  | 2005-2009 | 2 |
|  | 2010-2014 | 1 |
|  | 2015-2019 | 0 |
| **Disease stage** | Localized | 0 |
|  | Regional | 42 |
|  | Distant | 73 |
| **Primary tumor site** | Uterus | 31 |
|  | Ovary | 19 |
|  | Lung and bronchus | 31 |
|  | Breast | 27 |
|  | Urinary bladder | 28 |
|  | Peritoneum, omentum and mesentery | 26 |
|  | Soft tissue including heart | 49 |
|  | Salivary gland | 3 |
|  | Other non-epithelial skin | 0 |
|  | Gallbladder | 53 |
| **Treatment** | No treatment/Unknown | 100 |
|  | Single treatment, chemotherapy | 43 |
|  | Single treatment, radiotherapy | 76 |
|  | Single treatment, surgery | 43 |
|  | Chemotherapy+ radiotherapy | 45 |
|  | Chemotherapy+ surgery | 14 |
|  | Radiotherapy+ surgery | 26 |
|  | Chemotherapy+ radiotherapy+ surgery | 0 |
| **1-year survival probability** | | |
|  | Survival probability | Total score |
|  | 0.1 | 205 |
|  | 0.2 | 188 |
|  | 0.3 | 174 |
|  | 0.4 | 160 |
|  | 0.5 | 147 |
|  | 0.6 | 132 |
|  | 0.7 | 115 |
|  | 0.8 | 92 |
|  | 0.9 | 56 |
| **3-year survival probability** | | |
|  | Survival probability | Total score |
|  | 0.1 | 164 |
|  | 0.2 | 147 |
|  | 0.3 | 133 |
|  | 0.4 | 120 |
|  | 0.5 | 106 |
|  | 0.6 | 91 |
|  | 0.7 | 74 |
|  | 0.8 | 51 |
|  | 0.9 | 15 |
| **5-year survival probability** |  |  |
|  | Survival probability | Total score |
|  | 0.1 | 150 |
|  | 0.2 | 133 |
|  | 0.3 | 119 |
|  | 0.4 | 106 |
|  | 0.5 | 92 |
|  | 0.6 | 77 |
|  | 0.7 | 60 |
|  | 0.8 | 37 |
